# Gene polymorphisms in the PI3K/AKT/mTOR signaling pathway contribute to prostate cancer susceptibility in Chinese men

**DOI:** 10.18632/oncotarget.18064

**Published:** 2017-05-22

**Authors:** Ting Liu, Abulajiang Gulinaer, Xiaoli Shi, Feng Wang, Hengqing An, Wenli Cui, Qiaoxin Li

**Affiliations:** ^1^ Department of Pathology, The First Affiliated Hospital, Xinjiang Medical University, Urumqi, China; ^2^ Department of Urology, The First Affiliated Hospital, Xinjiang Medical University, Urumqi, China

**Keywords:** case-control study, prostate cancer, genetic susceptibility, PI3K/AKT/mTOR pathway, polymorphism

## Abstract

In this hospital-based case-control study of 413 prostate cancer (PCa) cases and 807 cancer-free controls, we investigated the role of functional single nucleotide polymorphisms (SNPs) of pivotal genes in the PI3K/AKT/mTOR pathway. We genotyped 17 SNPs in *mTOR*, *Raptor, AKT1*, *AKT2*, *PTEN*, and *K-ras* and found that 4 were associated with PCa susceptibility. Among the variants, the homozygote variant CC genotype of *mTOR* rs17036508 C>T were associated with higher PCa risk than the wild TT genotypes (adjusted OR = 3.73 (95% CI = 1.75-7.94), *P* = 0.001). The GT genotype of *mTOR* rs2295080 G>T was more protective than the TT genotypes (adjusted OR=0.54 (95% CI=0.32-0.91), *P=*0.020). The distributions of *Raptor* rs1468033 A>G genotypes differed between cases and controls, especially in subgroups defined by age, BMI, smoking status, and ethnicity. The CT/CC genotypes of *AKT2* rs7250897 C>T were associated with an increased risk of PCa, particularly in subgroups of age >71 and BMI >24 kg/m^2^. These findings suggest that SNPs in the PI3K/AKT/mTOR pathway may contribute to the risk of PCa in Chinese men.

## INTRODUCTION

Prostate cancer (PCa) is the leading malignancy in developed nations and ranks second in cancer deaths worldwide [1, 2]. Its incidence has increased rapidly in China [3, 4], with an estimated incidence of 1 in 10000 in 2010 [5]. Although environmental and genetic factors are associated with PCa carcinogenesis, the actual causes are unknown. Published genome-wide association studies (GWAS) identified 40 single nucleotide polymorphisms (SNPs) that were associated with human PCa risk [6]. Lu *et al.* demonstrated that SNPs associated with PCa risk were enriched in the androgen receptor (AR)-binding sites [7, 8]. These findings led to further functional studies to better understand PCa susceptibility.

The PI3K/AKT/mTOR signaling pathway is involved in many human malignancies, including PCa [9–11]. Nearly 25-70% of PCa cases show altered PI3K/AKT/mTOR signaling with higher prevalence in metastatic tumors. AKT is a proto-oncogene that is phosphorylated by a variety of extracellular stimuli and regulates multiple cellular processes involved in cell survival, growth, differentiation and proliferation. *G*enomic mutations of *PTEN* are reported for nearly 50% of primary PCa, especially in advanced disease [12]. Although AR is not a known physiological substrate of AKT, two AKT phosphorylation consensus sequences were identified in AR [13, 14]. It was postulated that some AKT substrate, eukaryotic initiation factor 4E-binding protein-1 (4EBP1) and ribosomal protein S6 kinase (P70) were associated with AR synthesis and resulted in PCa. Given these findings, the role of PI3K/AKT/mTOR pathway in PCa carcinogenesis needs to be established.

Previous pre-GWAS studies demonstrated that genetic variants in PI3K/AKT/mTOR pathway were associated with cancer risk, including PCa [15, 16]. However, they needed to be experimentally verified [7, 8]. In the present study, we analyzed if 17 SNPs in six pivotal genes (*K-ras*, *PTEN*, *AKT1*, *AKT2*, *mTOR*, and *Raptor*) of the mTOR pathway were associated with PCa susceptibility.

## RESULTS

### Characteristics of the subjects

Overall, there were no differences in distributions of age, smoking status, and BMI index between the 413 cases and 807 cancer-free controls (Table 1). Among the case subjects, 321 (77.7%) Han and 92 (22.3%) Uygur case subjects were included. Of those, 221 (53.5%) cases had Gleason scores <8 and the remaining 192 (46.5%) had Gleason scores ≥ 8.

**Table 1:** Distribution of demographic and clinico-pathological characteristics of prostate cancer patients and cancer-free controls from Chinese men

Variables	Cases no. (%)	Controls no. (%)	*P*^a^
All subjects	413 (100)	807 (100)	
Age, yr (Mean ± SD)	72 ±7.59	72 ±7.65	0.713
≤71	171 (41.4)	343 (42.5)	
>71	242 (58.6)	464 (57.5)	
Ethnic group			0.179
Han	321 (77.7)	599 (74.2)	
Uygur	92 (22.3)	208 (25.8)	
BMI (kg/m^2^)			0.346
≤24	217 (52.5)	401 (49.7)	
>24	196 (47.5)	406 (50.3)	
Smoking status			0.452
Never	190 (46.0)	353 (43.7)	
Ever	223 (54.0)	454 (56.3)	
Gleason score			
<8	221 (53.5)		
>=8	192 (46.5)		

SD: standard deviation; BMI: body mass index.

^a^Two-sided chi-square tests were used to calculate differences in the frequency distribution of genotypes between cases and controls.

### Genotype distributions and their association with PCa risk

Among the 17 SNPs analyzed, three variants of two genes were associated with PCa risk, and another variant was associated with PCa risk by stratification analysis (Table 2). The genotype distributions for *mTOR* rs17036508 C>T (*P*=0.001) and rs2295080 G>T (*P*=0.048) were different between cases and controls. The homozygote variant genotypes CC of *mTOR* rs17036508 C>T were associated with PCa risk compared with genotypes TT (adjusted OR=3.73 (95% CI=1.75-7.94), *P=*0.001). Also, heterozygote genotypes GT of *mTOR* rs2295080 G>T were protective compared to homozygote genotypes TT (adjusted OR=0.54 (95% CI= 0.32-0.91), *P* = 0.020). Furthermore, variants rs17036508 C>T [additive: adjusted OR=1.31 (95% CI =1.04-1.65), *P*=0.023; recessive: adjusted OR=3.69 (95% CI =1.74-7.83), *P*=0.001] and rs2295080 G>T [dominant: adjusted OR=0.59 (95% CI = 0.36-0.96), *P*=0.035] were also associated with PCa risk. For *Raptor* variants, heterozygote genotypes AG of polymorphism rs1468033 A>G were associated with PCa risk compared to genotypes GG (adjusted OR=1.61 (95% CI =1.25-2.06), *P* <0.001). We also found that rs1468033 A>G was associated with PCa risk [additive model: adjusted OR=1.42 (95% CI =1.17-1.73), *P*<0.001; dominant model: adjusted OR=1.61 (95% CI = 1.26-2.05), *P*<0.001]. Further analysis of the combined genotypes of these three SNPs and *AKT2* rs7250897 C>T showed enhanced PCa susceptibility with increasing numbers of putative high-risk genotypes (*P*_trend_<0.001) (Table 3).

**Table 2:** Logistic regression analysis of associations between genotypes of PI3K/AKT/mTOR genes and prostate cancer risk in Chinese men

Variables (HWE)^a^	Cases (N=1004)	Controls (N=1051)	*P*^b^	Crude OR (95% CI)	*P*	Adjusted OR (95% CI)^b^	*P*^c^
***mTOR* rs1034528 HWE:0.131**							
GG	260(63.0)	507(62.8)		1.00		1.00	
CG	132(32.0)	274(34.0)		0.94(0.73-1.21)	0.632	0.93(0.72-1.20)	0.563
CC	21(5.1)	26(3.2)		1.58(0.87-2.85)	0.134	1.59(0.87-2.90)	0.129
Additive model			0.248	1.06(0.86-1.30)	0.612	1.05(0.85-1.30)	0.656
Dominant model			0.965	1.00(0.0.78-1.27)	0.965	0.98(0.77-1.26)	0.895
Recessive model			0.110	1.61(0.89-2.90)	0.113	1.63(0.90-2.96)	0.105
***mTOR* rs17036508 HWE:0.451**							
TT	299(72.4)	610(75.6)		1.00		1.00	
CT	94(22.8)	186(23.1)		1.03(0.78-1.37)	0.744	1.05(0.79-1.40)	0.744
CC	20(4.8)	11(1.4)		**3.71(1.76-7.84)**	**0.001**	**3.73(1.75-7.94)**	**0.001**
Additive model			**0.001**	**1.29(1.03-1.63)**	**0.029**	**1.31(1.04-1.65)**	**0.023**
Dominant model			0.226	1.18(0.90-1.55)	0.226	1.20(0.92-1.58)	0.188
Recessive model			**<0.001**	**3.68(1.75-7.76)**	**0.001**	**3.69(1.74-7.83)**	**0.001**
***mTOR* rs12122605 HWE:0.533**							
CC	249(60.3)	488(60.5)		1.00		1.00	
CT	142(34.4)	283(35.1)		0.98(0.76-1.27)	0.897	0.98(0.76-1.26)	0.871
TT	22(5.3)	36(4.5)		1.20(0.69-2.08)	0.522	1.22(0.70-2.14)	0.479
Additive model			0.791	1.03(0.84-1.26)	0.767	1.03(0.84-1.27)	0.755
Dominant model			0.951	1.01(0.79-1.28)	0.951	1.01(0.79-1.29)	0.961
Recessive model			0.501	1.21(0.70-2.08)	0.502	1.23(0.71-2.14)	0.456
***mTOR* rs2295080 HWE:0.085**							
TT	236(57.1)	454(56.3)		1.00		1.00	
GT	145(35.1)	316(39.2)		**0.53(0.32-0.89)**	**0.015**	**0.54(0.32-0.91)**	**0.020**
GG	32(7.8)	37(4.6)		0.60(0.37-1.00)	0.050	0.62(0.38-1.03)	0.064
Additive model			**0.048**	0.94(0.77-1.14)	0.532	0.95(0.78-1.16)	0.633
Dominant model			**0.024**	**0.58(0.35-0.93)**	**0.025**	**0.59(0.36-0.96)**	**0.035**
Recessive model			0.768	1.04(0.82-1.32)	0.768	1.05(0.83-1.34)	0.676
***Raptor* rs1468033 HWE:0.278**							
GG	165(34.0)	415(51.4)		1.00		1.00	
AG	217(52.5)	336(41.6)		**1.62(1.27-2.08)**	**<0.001**	**1.61(1.25-2.06)**	**<0.001**
AA	31(7.5)	56(6.9)		1.39(0.87-2.24)	0.172	1.61(0.99-2.62)	0.053
Additive model			**0.001**	**1.37(1.13-1.65)**	**0.001**	**1.42(1.17-1.73)**	**<0.001**
Dominant model			**<0.001**	**1.59(1.25-2.02)**	**<0.001**	**1.61(1.26-2.05)**	**<0.001**
Recessive model			0.716	1.09(0.69-1.72)	0.716	1.27(0.80-2.02)	0.317
***Raptor* rs2271610 HWE:0.558**							
GG	243(58.8)	497(61.6)		1.00		1.00	
CG	140(33.9)	269(33.3)		1.06(0.82-1.37)	0.632	1.07(0.82-1.38)	0.640
CC	30 (7.3)	41(5.1)		1.50(0.91-2.46)	0.111	1.64(0.99-2.70)	0.054
Additive model			0.272	1.14(0.94-1.39)	0.177	1.17(0.96-1.43)	0.117
Dominant model			0.352	1.12(0.88-1.43)	0.353	1.14(0.89-1.45)	0.299
Recessive model			0.123	1.46(0.90-2.38)	0.125	1.60(0.98-2.62)	0.062
***Raptor* rs2271612 HWE:0.138**							
CC	185(44.8)	346(42.9)		1.00		1.00	
CT	182(44.1)	350(43.4)		0.97(0.76-1.25)	0.829	0.98(0.76-1.26)	0.861
TT	46(11.1)	111(13.8)		0.78(0.53-1.14)	0.197	0.76(0.52-1.13)	0.174
Additive model			0.424	0.91(0.76-1.08)	0.274	0.90(0.76-1.08)	0.259
Dominant model			0.522	0.93(0.73-1.18)	0.522	0.93(0.73-1.18)	0.527
Recessive model			0.197	0.79(0.55-1.13)	0.197	0.77(0.53-1.12)	0.169
***Raptor* rs2292639 HWE:0.085**							
CC	129(31.2)	235(29.1)		1.00		1.00	
AC	202(48.9)	394(48.8)		0.93(0.71-1.23)	0.625	0.94(0.71-1.24)	0.645
AA	82(19.9)	178(22.1)		0.84(0.60-1.18)	0.310	0.86(0.61-1.21)	0.376
Additive model			0.597	0.92(0.78-1.09)	0.315	0.93(0.78-1.10)	0.378
Dominant model			0.445	0.90(0.70-1.17)	0.445	0.91(0.70-1.18)	0.490
Recessive model			0.374	0.88(0.65-1.17)	0.374	0.89(0.66-1.20)	0.450
***Raptor* rs3751932 HWE:0.541**							
CC	290(20.2)	562(69.6)		1.00		1.00	
CT	112(27.1)	220(27.3)		0.99(0.76-1.29)	0.921	0.99(0.0.76-1.30)	0.966
TT	11(2.7)	25(3.1)		0.85(0.41-1.76)	0.666	0.90(0.43-1.86)	0.773
additive model			0.909	0.97(0.77-1.21)	0.753	0.98(0.78-1.23)	0.848
Dominant model			0.835	0.97(0.75-1.26)	0.836	0.99(0.76-1.28)	0.907
Recessive model			0.671	0.86(0.42-1.76)	0.672	0.90(0.44-1.86)	0.775
***Raptor* rs3751934 HWE:0.885**							
CC	136(32.9)	278(34.5)		1.00		1.00	
AC	200(48.4)	393(48.7)		1.04(0.80-1.36)	0.772	1.02(0.78-1.33)	0.912
AA	77 (18.6)	136 (16.9)		1.16(0.82-1.64)	0.409	1.12(0.79-1.59)	0.523
Additive model			0.708	1.07(0.90-1.27)	0.433	1.05(0.89-1.25)	0.563
Dominant model			0.596	1.07(0.83-1.38)	0.596	1.04(0.81-1.34)	0.748
Recessive model			0.435	1.13(0.83-1.54)	0.436	1.11(0.81-1.52)	0.509
***AKT1* rs2494750 HWE:0.165**							
GG	128(31.0)	240(29.7)		1.00		1.00	
CG	196(47.5)	382(47.3)		0.96(0.73-1.27)	0.783	0.97(0.74-1.28)	0.831
CC	89(21.6)	185(22.9)		0.90(0.65-1.26)	0.542	0.89(0.64-1.24)	0.484
Additive model			0.830	0.95(0.81-1.12)	0.547	0.94(0.80-1.11)	0.497
Dominant model			0.652	0.94(0.73-1.22)	0.651	0.94(0.73-1.22)	0.657
Recessive model			0.586	0.92(0.69-1.23)	0.586	0.90(0.68-1.21)	0.493
***AKT1* rs2494752 HWE:0.122**							
AA	161(39.0)	305(37.8)		1.00		1.00	
AG	189(45.8)	365(45.2)		0.98(0.76-1.27)	0.884	1.01(0.78-1.32)	0.921
GG	63(15.3)	137(17.0)		0.87(0.61-1.24)	0.445	0.87(0.61-1.25)	0.459
Additive model			0.736	0.94(0.80-1.12)	0.496	0.95(0.80-1.13)	0.558
Dominant model			0.686	0.95(0.75-1.21)	0.685	0.98(0.76-1.25)	0.837
Recessive model			0.442	0.88(0.64-1.22)	0.442	0.87(0.63-1.20)	0.396
***AKT2* rs2304186 HWE:0.036**							
GG	120(29.1)	224(27.8)		1.00		1.00	
GT	209(50.6)	430(53.3)		0.91(0.69-1.20)	0.491	0.91(0.69-1.20)	0.493
TT	84(20.3)	153(19.0)		1.03(0.73-1.45)	0.890	1.00(0.71-1.43)	0.981
Additive model			0.669	1.00(0.84-1.19)	0.984	0.99(0.83-1.18)	0.932
Dominant model			0.633	0.94(0.72-1.22)	0.633	0.93(0.72-1.22)	0.605
Recessive model			0.564	1.09(0.81-1.47)	0.564	1.07(0.79-1.44)	0.660
***AKT2* rs7250897 HWE:0.106**							
TT	181(43.8)	397(49.2)		1.00		1.00	
CT	190(46.0)	324(40.2)		1.29(1.00-1.65)	0.049	1.29(1.00-1.67)	0.047
CC	42(10.2)	86(10.7)		1.07(0.71-1.61)	0.742	1.11(0.73-1.67)	0.631
Additive model			0.139	1.12(0.94-1.33)	0.226	1.13(0.94-1.35)	0.183
Dominant model			0.633	1.24(0.98-1.58)	0.076	1.25(0.99-1.60)	0.066
Recessive model			0.564	0.95(0.64-1.40)	0.794	0.98(0.66-1.45)	0.909
***AKT2* rs7254617 HWE:0.120**							
GG	298(72.2)	581(72.0)		1.00		1.00	
AG	102(24.7)	214(26.5)		0.93(0.71-1.22)	0.600	0.93(0.71-1.23)	0.619
AA	13(3.2)	12(1.5)		2.11(0.95-4.69)	0.066	2.00(0.90-4.47)	0.091
Additive model			0.134	1.06(0.84-1.34)	0.621	1.06(0.83-1.34)	0.654
Dominant model			0.953	0.99(0.76-1.29)	0.953	0.99(0.76-1.29)	0.949
Recessive model			0.053	2.15(0.97-4.76)	0.058	2.04(0.91-4.54)	0.082
***PTEN* rs701848 HWE:0.212**							
TT	134(32.5)	245(30.4)		1.00		1.00	
CT	210(50.9)	415(51.4)		0.93(0.71-1.21)	0.570	0.93(0.71-1.22)	0.588
CC	69(16.7)	147(18.2)		0.86(0.60-1.22)	0.399	0.87(0.61-1.25)	0.460
Additive model			0.687	0.93(0.78-1.10)	0.386	0.93(0.78-1.11)	0.442
Dominant model			0.456	0.91(0.70-1.17)	0.456	0.91(0.71-1.18)	0.492
Recessive model			0.514	0.90(0.66-1.23)	0.514	0.92(0.67-1.26)	0.584
***K-ras* rs7312175 HWE:0.127**							
GG	294(71.2)	592(73.4)		1.00		1.00	
AG	210(25.9)	192(23.8)		1.12(0.85-1.48)	0.411	1.18(0.90-1.56)	0.238
AA	12(2.9)	23(2.9)		1.05(0.52-2.14)	0.892	1.07(0.52-2.19)	0.860
Additive model			0.712	1.09(0.87-1.36)	0.478	1.13(0.90-1.42)	0.314
Dominant model			0.421	1.12(0.86-1.45)	0.421	1.17(0.90-1.53)	0.250
Recessive model			0.956	1.02(0.50-2.07)	0.956	1.02(0.50-2.09)	0.952

OR: odds ratio; CI: confidence interval.

^a^Hard-Wenberg equilibrium test for controls.

^b^Two-sided Chi-square tests were used to calculate differences in the frequency distribution of genotypes between cases and controls.

^c^Adjusted for age, smoking, and BMI status in logistic regress models.

The results were in bold, if the 95% CI excluded 1 or *P*<0.05.

**Table 3:** Combined effects of risk genotypes of of PI3K/AKT/mTOR genes by dominant genetic models

Variables genotypes	Cases (N=413)	Controls (N=807)	*P*^a^	Crude OR (95% CI)	*P*	Adjusted OR (95% CI)^a^	*P*^b^
0	33(8.0)	142(17.6)	**<0.001**	1.00		1.00	
1	114(27.6)	251(31.1)		**1.95(1.26-3.03)**	**0.003**	**2.04(1.31-3.18)**	**0.002**
2	178 (43.1)	226 (28.0)		**3.39(2.21-5.19)**	**<0.001**	**3.50(2.27-5.38)**	**<0.001**
3	86 (20.8)	188(23.3)		**1.97(1.25-3.11)**	**0.004**	**2.07(1.30-3.28)**	**0.002**
4	2(0.5)	0(0)		**——**		**——**	
						*P*_trend_<0.001	
0	33(8.0)	142(17.6)	**<0.001**	1.00		1.00	
≥1	380(92.0)	665(82.4)		**2.46(1.65-3.67)**	**<0.001**	**2.56(1.71-3.84)**	**<0.001**

^a^Chi-square test was used to calculate the genotype frequency distributions.

^b^Obtained under dominant models in logistic regression analyses with adjustment for age, smoking status and BMI.

The results were in bold, if the 95% CI excluded 1 or *P*<0.05.

### Stratification analysis of PCa risk associated with significant variants

The *mTOR* rs17036508 CT/CC genotypes correlated with increased PCa risk particularly in subgroup of age ≤71 (dominant model: adjusted OR =1.81 (95% CI =1.19-2.77), *P* = 0.006). Further, the *mTOR* rs17036508 CC genotypes were associated with increased PCa risk by recessive genetic model for subgroups of age ≤71(adjusted OR=4.75 (95%CI =1.78-12.71), *P* = 0.002), age >71 (adjusted OR=4.88 (95%CI =1.65-14.38), *P* = 0.004), BMI ≤24 kg/m^2^ (adjusted OR=3.20 (95%CI =1.2-8.53), *P* = 0.02), BMI >24 kg/m^2^ (adjusted OR=8.11 (95%CI =2.62-25.11), *P*< 0.001), ever smokers (adjusted OR=6.39 (95%CI =2.46-16.6), *P*< 0.001), and Uygur population (adjusted OR=5.09 (95%CI =2.2-11.78), *P*< 0.001). On the contrary, the *mTOR* rs2295080 GT/GG genotypes were associated with decreased PCa risk by a dominant genetic model in BMI >24 kg/m^2^ and ever smokers subgroups. However, the *mTOR* rs2295080 GG genotypes were associated with PCa risk among age subgroups according to the recessive genetic model. For *Raptor* rs1468033 A>G, AG/AA genotypes were associated with increased PCa risk by the dominant genetic model, particularly in subgroups of age >71(adjusted OR=1.81 (95%CI =1.31-2.48), *P* = 0.003), BMI >24kg/m^2^ (adjusted OR=2.02 (95%CI =1.42-2.87), *P*< 0.001), never smokers (adjusted OR=1.61 (95%CI =1.21-2.30), *P* = 0.009), ever smokers (adjusted OR=1.58 (95%CI =1.13-2.19), *P* = 0.068), and Uygur population (adjusted OR=1.66 (95%CI =1.26-2.20), *P*< 0.001). Furthermore, increased PCa risk was observed by recessive genetic model for the BMI >24 kg/m^2^ subgroup (adjusted OR=5.13 (95% CI=1.94-13.59), *P* = 0.001). Increased PCa risk was observed for *AKT2* rs7250897 C>T by the dominant genetic model, particularly in subgroups of age >71(adjusted OR=1.83 (95%CI =1.33-2.52), *P* = 0.002) and BMI >24kg/m^2^ (adjusted OR=1.58 (95%CI =1.12-2.24), *P* = 0.010). However, further homogeneity tests showed no difference in risk estimates between subgroups for most strata except age group by *mTOR* rs17036508 CT/CC genotypes (*P*=0.032), *mTOR* rs2295080 GG genotypes (*P*=0.002), *AKT2* rs7250897 CT/CC genotypes (*P*<0.001) and BMI group by *Raptor* rs1468033 AA genotypes (*P*<0.001). The details are shown in Table 4.

**Table 4:** Stratification analysis for associations between PI3K/AKT/mTOR variants and prostate cancer risk in Chinese men

Variables	*mTOR* rs17036508		Adjusted	*P*^a^	*P*^hom^	*mTOR* rs2295080		Adjusted	*P*^a^	*P*^hom^	*Raptor* rs1468033		Adjusted	*P*^a^	*P*^hom^	*AKT2* rs7250897		Adjusted	*P*^a^	*P*^hom^
	(Cases/Controls)		OR (95%CI)^a^			(Cases/Controls)		OR (95%CI)^a^			(Cases/Controls)		OR (95%CI)^a^			(Cases/Controls)		OR (95%CI)^a^		
By DOM	CT+CC	TT				GT+GG	TT				AG+AA	GG				CT+CC	TT			
Age, yr (median)																				
≤71	53/69	118/274	**1.81** **(1.19-2.77)**	**0.006**	**0.032**	158/329	13/14	0.54 (0.25-1.19)	0.127	0.741	102/180	69/163	1.38(0.94-2.01)	0.097	0.227	82/191	89/152	0.76 (0.52-1.10)	0.145	**<0.001**
>71	66/128	176/336	1.02 (0.72-1.45)	0.926		223/441	19/23	0.60 (0.32-1.14)	0.120		146/212	96/252	**1.81(1.31-2.48)**	**<0.001**		150/219	92/245	**1.83** **(1.33-2.52)**	**<0.001**	
BMI, kg/m^2^																				
≤24	63/105	154/296	1.23 (0.85-1.79)	0.280	0.536	203/382	14/19	0.70 (0.34-1.45)	0.339	0.366	123/203	94/198	1.25(0.89-1.75)	0.196	0.062	114/212	103/189	0.99 (0.71-1.39)	0.959	0.051
>24	56/92	140/314	1.36 (0.93-1.01)	0.117		178/388	18/18	**0.43** **(0.22-0.86)**	**0.016**		125/189	71/217	**2.02(1.42-2.87)**	**<0.001**		118/198	78/208	**1.58** **(1.12-2.24)**	**0.010**	
Smoking status																				
Never	50/80	140/273	1.22 (0.81-1.84)	0.341	0.835	179/335	11/18	0.87 (0.40-1.89)	0.728	0.152	113/169	77/184	**1.61(1.12-2.30)**	**0.009**	0.982	102/171	88/182	1.24 (0.87-1.77)	0.231	0.938
Ever	69/117	154/337	1.34 (0.94-1.92)	0.109		202/435	21/19	**0.42** **(0.22-0.80)**	**0.008**		135/223	88/231	**1.58(1.13-2.19)**	**0.007**		130/239	93/215	1.24 (0.89-1.72)	0.206	
Ethnic group																				
Han	24/52	68/156	1.12 (0.63-1.97)	0.703	0.504	82/194	10/14	0.61 (0.26-1.45)	0.266	0.871	51/96	41/112	1.44(0.87-2.36)	0.155	0.693	51/94	41/114	1.52 (0.92-2.51)	0.099	0.358
Uygur	95/145	226/454	1.33 (0.98-1.81)	0.068		299/576	22/23	0.56 (0.30-1.02)	0.057		197/296	124/303	**1.66(1.26-2.20)**	**<0.001**		181/316	140/283	1.18 (0.90-1.56)	0.237	
**Variables**	***mTOR*** **rs17036508**		**Adjusted**	***P***^a^	***P***^hom^	***mTOR*** **rs2295080**		**Adjusted**	***P***^a^	***P***^hom^	***Raptor*** **rs1468033**		**Adjusted**	***P***^a^	***P***^hom^	***AKT2*** **rs7250897**		**Adjusted**	***P***^a^	***P***^hom^
	**(Cases/Controls)**		**OR** **(95%CI)**^a^			**(Cases/Controls)**		**OR** **(95%CI)**^a^			**(Cases/Controls)**		**OR** **(95%CI)**^a^			**(Cases/Controls)**		**OR** **(95%CI)**^a^		
**by REM**	CC	TT+CT				GG	TT+GT				AA	GG+AG				CC	TT+CT			
Age, yr (median)																				
≤71	157/337	14/6	**4.75** **(1.78-12.71)**	**0.002**	0.854	81/128	90/215	**0.67** **(0.46-0.97)**	**0.035**	**0.002**	158/318	13/25	1.19(0.54-2.42)	0.635	0.882	154/303	17/40	0.89 (0.49-1.63)	0.710	0.577
>71	231/459	11/5	**4.88** **(1.65-14.38)**	**0.004**		96/225	146/239	**1.46** **(1.06-2.00)**	**0.021**		224/433	18/31	1.34(0.72-2.49)	0.351		217/418	25/46	1.05 (0.62-1.76)	0.858	
BMI, kg/m^2^																				
≤24	206/394	11/7	**3.20** **(1.20-8.53)**	**0.020**	0.210	90/176	127/225	1.09 (0.78-1.53)	0.622	0.592	200/351	17/50	0.66(0.37-1.18)	0.163	**<0.001**	193/352	24/49	0.89 (0.53-1.51)	0.674	0.943
>24	182/402	14/4	**8.11** **(2.62-25.11)**	**<0.001**		87/177	109/229	0.97 (0.69-1.37)	0.859		182/400	14/6	**5.13(1.94-13.6)**	**0.001**		178/369	18/37	0.99 (0.55-1.80)	0.985	
Smoking status																				
Never	182/348	8/5	3.01 (0.97-9.34)	0.057	0.352	81/157	109/196	1.08 (0.76-1.55)	0.663	0.775	180/333	10/20	0.94(0.43-2.07)	0.884	0.588	170/319	20/34	1.11 (0.62-1.99)	0.729	0.507
Ever	206/448	17/6	**6.39** **(2.46-16.60)**	**<0.001**		96/196	127/258	1.00 (0.72-1.39)	0.987		202/418	21/36	1.45(0.82-2.59)	0.206		201/402	22/52	0.86 (0.51-1.47)	0.589	
Ethnic group																				
Han	87/205	5/3	3.90 (0.90-16.98)	0.069	0.794	37/89	55/119	1.08 (0.65-1.78)	0.778	0.764	85/191	7/17	1.10(0.43-2.80)	0.848	0.675	84/188	8/20	0.95 (0.40-2.26)	0.902	0.893
Uygur	301/591	20/8	**5.09** **(2.20-11.78)**	**<0.001**		140/264	181/335	1.05 (0.79-1.38)	0.747		297/560	24/39	1.34(0.78-2.30)	0.284		287/533	34/66	0.99 (0.63-1.53)	0.946	

BMI: body mass index. ^a^ Obtained under dominant models in logistic regression analyses with adjustment for age, smoking status and BMI. ^b,c^ According to the current WHO recommendations.

*P*^hom^*P* value for homogeneiy test. DOM: dominant genetic model; REM: recessive genetic model.

The results were in bold, if *P*<0.05.

### Association of high-order interactions with PCa risk

We performed the multifactor dimensionality reduction (MDR) analysis by including the genotypes of four significant genetic factors (*mTOR* rs17036508 CC and rs2295080 TT, *Raptor* rs1468033 AG/AA, and *AKT2* rs7250897 TT/CT) and four environmental risk factors (age at diagnosis, smoking status, race, and BMI). Among all 8 factors, the *Raptor* rs1468033 A>G variant was the best one-factor model. Likewise, interactions between *mTOR* rs17036508 CC, rs2295080 TT and *Raptor* rs1468033 AG/AA represented the best three-factor model. Age was the most promising environmental risk factor associated with the four genetic factors. The details are presented in Table 5. Subsequent hierarchical cluster analysis placed BMI and smoking status, race and rs17036508 C>T, rs2295080 G>T and rs1468033 A>G, age and rs7250897 C>T on the same branch. This suggested that interactions in this eight-locus model may modulate PCa risk (data not shown).

**Table 5:** MDR analysis for the risk of prostate cancer prediction in an Chinese population

Best interaction models	Cross-validation	Average prediction error	*P*-value^a^
rs1468033	100/100	0.4566	0.0001
rs2295080 rs1468033	100/100	0.3451	p < 0.0001
rs17036508 rs2295080 rs1468033	**100/100**	**0.3434**	p < 0.0001
age rs17036508 rs2295080 rs1468033	99/100	0.4066	p < 0.0001
age rs17036508 rs2295080 rs1468033 rs7250897	78/100	0.4254	p < 0.0001
BMI smoking_status race rs17036508 rs2295080 rs1468033	45/100	0.4467	p < 0.0001
smoking_status age race rs17036508 rs2295080 rs1468033 rs7250897	61/100	0.4022	p < 0.0001
BMI smoking_status age race rs17036508 rs2295080 rs1468033 rs7250897	100/100	0.5066	p < 0.0001

MDR: multifactor dimensionality reduction.

The best model with maximum cross-validation consistency and minimum prediction error rate was in bold.

^a^*P*-value for 1000-fold permutation test.

Finally, we analyzed the false-positive report probability (FPRP) values at variant prior probability levels for all positive findings (Table 6). Some higher statistical power (81.7%-89.6%) was observed under the assumption of prior probability of 0.25. However, some significant findings were still noteworthy at prior probability of 0.01 in spite of the lower statistical power (range from 5% to 45%). Some findings with greater FPRP values need further validation in larger studies.

**Table 6:** False-positive report probability values for associations between the PCa risk and the frequency of Genotypes of PI3K/AKT/mTOR variants

Genotype	Crude OR (95%CI)	*P*^a^	Statistical power^b^	Prior probability				
				0.25	0.1	0.01	0.001	0.0001
**All patients**								
*mTOR* rs17036508 CC vs TT	3.71(1.76-7.84)	0.0006	0.008	**0.18**	0.397	0.879	0.986	0.999
*mTOR* rs17036508 CC vs CT/TT	3.68(1.74-7.76)	0.0003	0.005	**0.15**	0.345	0.853	0.983	0.998
*mTOR* rs2295080 GT vs TT	0.53(0.32-0.89)	0.0484	0.862	**0.144**	0.336	0.848	0.982	0.998
*mTOR* rs2295080 GG/GT vs TT	0.57(0.35-0.93)	0.0236	0.829	**0.079**	0.204	0.738	0.966	0.997
*raptor* rs1468033 AG vs GG	1.62(1.27-2.08)	0.0006	0.415	**0.004**	**0.013**	**0.125**	0.591	0.935
*raptor* rs1468033 AA/AG vs GG	1.59(1.25-2.02)	0.0001	0.284	**0.001**	**0.003**	**0.034**	0.26	0.779
***mTOR* rs17036508**								
Age≤71 yrs, CT/CC vs TT	1.69(1.11-2.57)	0.0143	0.363	**0.106**	0.262	0.796	0.975	0.997
Age≤71 yrs, CC vs CT/CC	4.24(1.56-11.50)	0.0022	0.61	**0.011**	**0.031**	0.263	0.783	0.973
Age>71 yrs,CC vs CT/CC	3.14(1.02-9.69)	0.0366	0.876	**0.111**	0.273	0.805	0.977	0.998
BMI<=24,CC vs CT/CC	2.72(1.02-7.24)	0.0378	0.896	**0.112**	0.275	0.807	0.977	0.998
BMI>24,CC vs CT/CC	5.40(1.67-17.45)	0.0017	0.571	**0.009**	**0.026**	0.228	0.748	0.968
Ever smoking, CC vs CT/TT	4.62(1.73-12.33)	0.0008	0.457	**0.005**	**0.016**	**0.148**	0.636	0.946
uygur, CC vs CT/TT	4.39(1.89-10.21)	0.0002	0.160	**0.004**	**0.011**	**0.110**	0.555	0.926
***mTOR* rs2295080**								
BMI>24, GG/GT vs TT	0.46(0.23-0.90)	0.0213	0.833	**0.071**	**0.187**	0.717	0.962	0.996
Ever smoking, GG/GT vs TT	0.42(0.22-0.80)	0.0067	0.722	**0.027**	**0.077**	0.479	0.903	0.989
Age≤71 yrs, GG vs GT/TT	0.66(0.46-0.96)	0.0288	0.504	**0.146**	0.34	0.85	0.983	0.998
Age>71 yrs, GG vs GT/TT	1.43(1.04-1.96)	0.0255	0.817	**0.086**	0.219	0.755	0.969	0.997
***Raptor* rs1468033**								
Age>71 yrs, AA/AG vs GG	1.81(1.32-2.48)	0.0002	0.276	**0.002**	**0.006**	**0.067**	0.42	0.879
BMI>24, AA/AG vs GG	2.02(1.42-2.87)	<0.0001	0.198	**0.002**	**0.005**	**0.048**	0.335	0.835
Ever smoking, AA/AG vs GG	1.59(1.15-2.20)	0.0051	0.547	**0.027**	**0.077**	0.48	0.903	0.989
Never smoking, AA/AG vs GG	1.60(1.12-2.28)	0.0099	0.712	**0.040**	**0.111**	0.579	0.933	0.993
Uygur group, AA/AG vs GG	1.63(1.23-2.14)	0.0005	0.433	**0.003**	**0.010**	**0.103**	0.536	0.920
BMI>24, AA vs AG/GG	5.13(1.94-13.56)	0.0003	0.382	**0.002**	**0.007**	**0.072**	0.440	0.887
***AKT2* rs7250897**								
Age>71 yrs, CT/CC vs TT	1.82(1.33-2.51)	0.0002	0.284	**0.002**	**0.006**	**0.065**	0.413	0.876
BMI>24, CT/CC vs TT	1.60(1.12-2.25)	0.0085	0.662	**0.037**	**0.104**	0.56	0.928	0.992
**Combined effect (risk genotype)**								
**0 vs >=1**	2.46(1.65-3.67)	<0.0001	0.050	**0.006**	**0.018**	**0.165**	0.666	0.952

OR: odds ratio; CI: confidence interval; BMI: body mass index.

^a^Chi-square test was used to calculate the genotype frequency distributions.

^b^Statistical power was calculated using the number of observations in the subgroup and the OR and *P* values in this table.

The results in false-positive report probability analysis were in bold, if the prior probability < 0.2.

## DISCUSSION

In the current single institution based case-control study, functional SNPs of six pivotal genes of PI3K/AKT/mTOR pathway were associated with PCa risk. Briefly, the *mTOR* rs17036508 CC, *mTOR* rs2295080 GG/GT, *Raptor* rs1468033 AG/AA, and *AKT* rs7250897 TT/CT genotypes were associated with PCa risk, especially in age, BMI, smoker-status, and ethnic subgroups. To the best of our knowledge, this is the first post-GWAS study analyzing associations of these six pivotal genes of PI3K/AKT/mTOR pathway with PCa risk.

The *mTOR* gene is a critical cellular protein with more than 2651 SNPs reported across the whole region [17]. However, only few SNPs have been associated with PCa susceptibility. Two studies showed that *mTOR* rs2295080 GT/GG genotypes protected against PCa risk in Han Chinese populations [18, 19]. These findings were consistent with mixed Chinese populations in the present study, but not in separate ethnic subgroups (Han or Uygur). The effects of the *mTOR* rs2295080 GT/GG genotypes were also observed in esophageal squamous cell carcinoma, gastric carcinoma, and renal cell carcinoma [20–22]. *In vitro* and *in vivo* studies suggested that *mTOR* rs2295080 T allele probably increased the affinity of special transcription factors to this promoter region and contributed to enhanced mTOR activity [20]. Variant *mTOR* rs2295080 was linked to eight potential functional variants of *mTOR* with higher linkage disequilibrium (LD) coefficient >0.8, including rs1064261, rs1074078, rs1135172, rs1883965, rs4845860, rs6540965, and rs6671083. Among these, the variant rs1064261 probably interrupted the exonic splicing enhancer or silencer motif and correlated with neuroendocrine tumors [23], gastric cancer [24], and esophageal squamous cell carcinoma [25]. Similarly, as a probable transcription factor binding site of *mTOR*, variant rs1883965 was associated with esophageal carcinoma, gastric cancer, and hepatocellular carcinoma [26]. Additionally, it was predicted that variant rs17036508 of *mTOR* was located within a miRNA binding site and an exonic splicing enhancer or silencer motif, thereby affecting pre-RNA splicing. The association between rs17036508 and PCa was also observed previously [19]. In the present study, we found that homozygote variant carriers of rs17036508 were more likely to develop cancer compared to homozygote wild carriers. In theory, polymorphism rs17036508 located in 3’-UTR of angiopoietin-like 7 gene (*ANGPTL7)*, resulting in upregulation of ANGPTL7 by hypoxia in cancer cells, which exerts a pro-angiogenesis effect [27]. Taken together, it is biologically plausible that these two polymorphisms might mediate tumor formation by regulating the expression of *mTOR* and *ANGPTL7* simultaneously.

The *Raptor* gene, located in 17q25.3 with 34 exons, regulates responses to nutrient and insulin levels [28]. The Raptor protein forms a stoichiometric complex with the mTOR kinase, and is associated with 4EBP1and P70. Previous *in vitro* and *in vivo* studies have shown that Raptor acts as a scaffold protein that regulates mTOR-dependent signaling [29]. One of the mechanisms involves changes in phosphorylation status of Raptor. However, the mechanism through which Raptor regulates these processes is only beginning to be established. In a recent study, *Raptor* gene polymorphisms were associated with increased risk for bladder cancer; physical activity, energy balance and genetic variants in the mTOR pathway co-coordinately influenced bladder cancer risk [30]. In this study, variant *Raptor* rs1468033 was associated with PCa risk, particularly in subgroups of age, BMI, and smoking status, similar to previous findings. *Silico* analysis indicated that rs1468033 was found with several potential functional polymorphisms of *Raptor* gene, including rs2292639, rs499609, rs6565500, and rs9899178. Among these, rs2292639, rs4999609, and rs6565500 were predicted as transfactor binding sites, whereas rs9899178 was predicted as splicing site. It is plausible that these variants modulate the expression of *Raptor* gene, and affect mTOR activity.

Although mTOR activity was previously implicated in promoting PCa cell invasion, the role of *AKT2* was not known. The different *AKT* isoforms (*AKT1*, *AKT2*, and *AKT3*) play contradictory regulatory roles; for example, AKT1 activation inhibits cell migration whereas AKT2 promotes it [31, 32]. Many studies have indicated that AKT2 is a more promising therapeutic target than PI3K due to its involvement in normal cellular processes. AKT2 downregulated GSK3b, which modulates cell migration [33]. Additionally, AKT negatively regulates Mdm2 expression during abnormal stress and promotes tumorigenesis [34]. Recently, Chen *et al.* reported that AKT2 rs7254617 increased prostate cancer risk [18]. Also, a Caucasian study showed that AKT2 variant rs3730050 was associated with poor prognosis of bladder cancer patients. Therefore, AKT isoforms are cancer susceptibility genes. In this study, *AKT2* rs7250897 was associated with increased PCa risk by a dominant genetic model in subgroups of age >71and BMI >24kg/m^2^, indicating that rs7250897 altered *AKT2* expression, which subsequently affected synthesis of adipose-related proteins. Since the three variants were far from each other, we postulated that they were associated with carcinogenesis by different mechanisms. These findings need to be further validated.

There were few limitations in the present study that need to be addressed. First, the limited sample size in our study may have decreased detection of weaker genetic effects in carcinogenesis. Second, information regarding predisposition to PCa was not collected for the analysis and might confuse the stratified positive associations. Third, the case-control study may have inherent selection and information biases. Because of insufficient medical records, we could not perform correlative analyses between stages of PCa and variants of AKT pathway that may have provided more information in PCa carcinogenesis. Moreover, further *in vitro* and *in vivo* experiments are necessary to unravel molecular mechanisms for the genetic associations that we have postulated in this study.

In summary, we showed that variants of PI3K/AKT/mTOR signal pathway genes may associate with PCa risk. The combined genotypes of these variants enhanced PCa risk with increasing numbers of putative high-risk genotypes. Moreover, the three-factor model (rs17036508, rs2295080, rs1468033) was the best model to predict PCa risk. In conclusion, our study postulated that the genetic variants may alter the expression and activity of *mTOR* leading to PCa susceptibility.

## MATERIALS AND METHODS

### Patients and controls

We recruited 413 newly diagnosed PCa cases and 807 frequency-matched cancer-free controls from genetically unrelated Chinese Han and Uygur participants in Xinjiang province between January 2003 and January 2015. The cases were histopathologically confirmed as primary prostate adenocarcinoma at the First Affiliated Hospital of Xinjiang medical University (XJMU). Pathological grades of the PCa were determined by Gleason scores from the radical prostatectomy specimens according to the latest WHO criteria [35]. The recruited healthy controls were extracted from males who had health check-up in the First Affiliated Hospital of XJMU during the same period. Individuals with serum PSA >4ng/ml were excluded from the control group.

All subjects were interviewed with a written informed consent. Response rate was 92% and 90% for cases and controls, respectively. The experimental and research protocols were approved by the Institutional Review Board of XJMU.

### Selection of single nucleotide polymorphisms

For the six pivotal genes in PI3K/AKT/mTOR pathway, selection strategy for the potentially functional SNPs was based on the NCBI dbSNP database (http://www.ncbi.nlm.nih.gov/projects/SNP) and SNPinfo web server (http://snpinfo.niehs.nih.gov/snpfunc.htm). The criteria were: 1) minor allele frequency (MAF) was at least 5% in Chinese populations; 2) SNPs were potentially functional according to SNPinfo prediction platform. Ultimately, 17 potentially functional variants were selected involving *mTOR* (rs1034528 C>G, rs1703658 C>T, rs12122605 T>C, and rs2295080 G>T), *Raptor* (rs2271610 C>G, rs2271612 T>C, rs2292639 A>C, rs3751932 T>C, rs3751934 A>C), *AKT1* (rs2494750 C>G, rs2494752 G>A), *AKT2* (rs2304186 T>G, rs7250897 C>T, rs7254617 A>G), *PTEN* (rs701848 C>T), and *K-ras* (rs7312175 A>G) based on the bioinformatics analysis performed with HaploView software 4.2. All these SNPs were genotyped by the TaqMan real-time PCR method as described previously [36]. In this study, the quality control strategy was established as follows: (1) the discrepancy rate in all positive controls was less than 0.1%; (2) the results with >95% call rates and 100% concordance for duplicated specimens were favorable for further analysis.

### Statistical analysis

We performed the Pearson’s χ^2^-test for the differences in selected variables between cases and controls. Crude and adjusted ORs and their 95% CIs were computed from both univariate and multivariate unconditional logistic regression models. We further evaluated the stratified associations based on the significant genetic models accompanied by the homogeneity Q-tests for the strata. For all the significant findings, we calculated FPRP with the assumption of different prior probabilities to detect any possible false positive associations. Only FPRP values<0.2 were considered noteworthy [37]. All statistical analyses were performed with SAS 9.1 statistical software (SAS, Cary, NC, USA) unless stated otherwise. All *P* values were two-sided with a significance level of *P*<0.05.

The multifactor dimensionality reduction (MDR) analysis was conducted by the MDR V2.0 beta 8.2 software (http://www.multifactordimensionalityreduction.org/) to identify the best *n*-factor interaction model [38]. We further performed the interaction dendrograms and graphs for risk loci of this study, and the color of branches and lines referred to the type of interaction, with green-to-yellow-to-red indicating weak-to-strong interactions.

## Abbreviations

SNPs: single nucleotide polymorphisms
PCa: prostate cancer
OR: odds ratio
CI: confidence interval
